# Problematic Social Networking Site use-effects on mental health and the brain

**DOI:** 10.3389/fpsyt.2022.1106004

**Published:** 2023-01-19

**Authors:** Aviv M. Weinstein

**Affiliations:** Psychology and Behavioral Science, Ariel University, Ariel, Israel

**Keywords:** problematic Social Network Site use, social media addiction, social media, fear of missing out, social networks

## Abstract

The association between excessive use of Social Networking Sites (SNS) and mental health is raising serious concern among health and education professionals. Problematic SNS use has been associated with an increased rate of depression, anxiety, stress, obsessive-compulsive disorder (OCD), attention-deficit/hyperactivity disorder (ADHD), and propensity to excessive alcohol use. It may also lead to vulnerability to aggression, cyberbullying and fear of missing out (FOMO). There is little evidence for cognitive impairments, but there is some preliminary event-related potentials (ERPs) evidence for inefficiency in allocating and monitoring resources and inhibitory control. Problematic SNS has been associated with the personality traits of conscientiousness agreeableness and neuroticism, and with narcissism. There is evidence for reduced sleep quality and quantity, longer sleeping latency and more sleep disturbance. The few brain imaging studies show some similarity between problematic SNS use and other addictions related to inhibitory-control mechanism, reduced gray matter volumes in the nucleus accumbens, amygdala, and the insula, suggesting rewarding effects of SNS use on the brain. Finally, there is preliminary evidence that treatment with Cognitive Behavior Therapy (CBT) can assist in short-term abstinence intervention to treat problematic SNS use. We conclude that problematic SNS use may have deleterious effects on emotional and social relationships, and more research is required on its effects on cognitive and brain function.

## Introduction

Over the past decade there has been a growing interest in the problematic use of social networking sites (SNSs) or Social media activity. The purpose of this narrative review is to summarize studies on problematic SNS use and Social media addiction from 2010 until now. Keywords were entered in a PubMed and Scopus search using “Problematic Social Networking Site use” and “Social media addiction” as search words and publications were limited from 2010 to October 2022. The search has yielded 797 records. These were screened for suitability by using inclusion criteria of English language, published in peer-reviewed journals, measure brain imaging in human subjects. Exclusion criteria were abstracts, dissertations, methodological papers and conference papers. Based on title and abstract, following these criteria, 699 records were excluded, and 98 records were included in this narrative review.

## The magnitude and definition of Problematic Social Networking Site use

There is a significant increase in the use of social networking sites (SNSs) or Social media activity. In 2021, over 4.26 billion people were using social media worldwide, a number projected to increase to almost six billion in 2027. Social media is an integral part of daily Internet use. On average, Internet users spend 144 minutes per day on social media and messaging applications, an increase of more than half an hour since 2015 (1). Facebook who leads the market, was the first to have over 1 billion accounts and it currently has more than 2.89 billion monthly users. The company also owns four companies Facebook, WhatsApp, Facebook Messenger, and Instagram with over 1 billion users each, and in 2021, Facebook reported over 3.58 billion monthly users (1). Given the extensive use of SNS, it is important to understand the potential risks involved in problematic SNS use. The World Health Organization (2) has raised Public health concerns over the similarity between signs and symptoms of Internet Gaming Disorder (IGD) including problematic SNS use, gambling disorder and substance use disorders.

Problematic SNS use has been defined by Andreassen and Pallesen (3) as “being highly concerned about SNSs, driven by a strong motivation to use SNSs, and to devote a lot of time and effort to SNSs that it impairs other social activities, education and or occupation, interpersonal relationships, and or psychological health and wellbeing.” There are different terms describing problematic SNS use, these include Social Media Use Disorder, or Social Media Disorder, and Networks Use Disorder, and the taxonomy of problems in the study of Internet use disorder is being discussed by Montag (4). The prevalence of problematic SNS varies among populations ranging from 1.6% in Nigeria (5), 4.5% in Hungary (6), 8.6% in Peru (7), and 12% in China (8), 8.9% among Italian adolescents (9). A meta-analysis has analyzed 63 independent samples of around 35,000 respondents from 32 nations has shown that prevalence rates varied among studies and the prevalence estimate was 5% for studies who used strict classifications (10).

## Comorbidity with other disorders

A recent review by Hussain and Griffiths (11) has shown a relationship between problematic SNS and depression, anxiety, stress, ADHD and OCD. Studies have shown a positive correlation between Problematic SNS use and depression (12–18) anxiety (12, 14, 16–19), ADHD and OCD (12) and stress (14, 19). Since then, new studies have found an association with depression (20–22) ADHD (23) and Social anxiety (24). Patients with major depressive disorder (MDD) were more addicted to SNS and “relapse” to problematic SNS use predicted depressive symptoms in these patients (25). A recent study has shown an association between problematic SNS use and eating disorder that was mediated by muscle dysmorphia (26). Problematic SNS use symptoms correlated with anxiety and narcissism (27, 28). Finally, problematic SNS use may place individuals at risk for potentially problematic drinking (29). Adolescents (age 11–13) in Italy, showed that problematic SNS use was linked with the propensity for substance use (30).

In summary, problematic SNS use is associated with mental health problems that are similar to those of an IGD, such as depression, anxiety (including social anxiety), OCD and ADHD and risk for problematic drinking.

## Emotional and social factors associated with problematic SNS use

### Low self-esteem and social anxiety

Low self-efficacy, positive outcome expectancies, and impulsivity have been identified in excessive SNS users (8). Among 8,912 college students across seven countries (U.S., Canada, Spain, England, Argentina, Uruguay, and South Africa) high ruminating thoughts have accounted for major depressive and social anxiety symptoms (31). Several studies have shown an association between low self-esteem (32, 33) and fear of negative self-evaluation (34) anxiety (35) and problematic SNS use.

### Cyberbullying, emotional abuse and distress

Problematic SNS use was linked with cyberbullying and cyber victimization of university students (36, 37). Students who reported that their upbringing style as inconsistent and unbalanced and those who showed an aggressive trait had higher scores of problematic SNS use (38). Child emotional abuse, indicated by deficient self-other differentiation and impaired reflective function was also associated with problematic SNS use (39). Stress, impulsiveness and reduced inhibitory control contributed to problematic SNS among lower socio-economic families in China (40). Finally, lower emotional intelligence predicted perceived stress, which contributed to depressive symptoms and problematic SNS use (41).

### Social comparison and “peer phubbing”

Problematic SNS use was also associated with social comparison which is linked with stress and impaired wellbeing) (42). “Peer phubbing” (the act of ignoring other people in the context of social contact by paying attention to his/her phone instead of focusing on the person directly in his/her company) correlated with problematic SNS use (43). Furthermore, social anxiety mediated the association between “peer phubbing” and problematic SNS use, particularly among undergraduates with family financial difficulties (43). Finally, “peer phubbing” was associated with loneliness and problematic SNS use (44).

### Emotion recognition and meta cognition deficits

Emotion recognition deficits among individuals with problematic SNS use were demonstrated on the Reading the Mind in the Eyes Test (RMET) (45). A following study has shown that faulty meta-cognitions (like worry, superstition, punishment, beliefs about responsibility, and cognitive monitoring) but not emotion recognition predicted problematic SNS use in adolescents (46).

### Body shame and body image

Female adolescents reported higher scores of body shame, social physique anxiety and problematic SNS use (47). Awareness and internalization mediated the association between body dissatisfaction and problematic SNS use (48). Depression and anxiety mediated the relationship between perceived stress and problematic SNS use and this relationship was moderated by psychological resilience, but not by social support (49). Negative body image correlated with frequency of SNS use and it was enhanced by exposure to appearance-related content on the Internet. Furthermore, negative body image or body shame was indirectly associated with adolescents' problematic SNS use (50). Finally, high levels of self-reflection was a protective factor against problematic SNS use among adolescents. Adolescents with problematic SNS use tend to have low exploration of self-identity and in crisis they consider alternative commitments instead (51).

### Depression

A meta-analysis has reported that depressive symptoms weakly correlated with time spent using SNS and intensity of SNS use (52). However, depressive symptoms moderately correlated with problematic SNS use, and it was not moderated by other factors like age or gender.

### External motivations of social reward

The urge to use SNS was associated with handling boredom rather than increase positive emotions or reduce negative emotions (53). Others stress the importance of social rewards like “likes,” social comparisons and connection with other people rather than motivations based on enjoyment and negative power (54). There is supporting evidence that young people tend to use SNSs to enhance their external expectations of having a large network size rather than internal expectations for subjective wellbeing (55). Finally, young adults who used social media for 2 h or more daily, increased perceived social isolation compared with those who use it for < 30 min each day (56). Similar associations were reported in middle-aged and older adults (57). It has been suggested that people who have reduced off-line social experiences and are highly influenced by social media tend to have unrealistic self-perceptions.

In summary, problematic SNS use has been associated with internal emotional factors like depression and anxiety, aggression and negative body image and external social motivations.

### Impaired cognitive and executive function

Emotional states and stress mediated the association between executive function, and problematic SNS use among Chinese female college students (58). There were no differences between problematic and non-problematic SNS use groups in cognitive flexibility and inhibitory control aspects of Executive Function measured by performance on the Wisconsin Card Sorting Test (WCST) (20). Categories achieved and number of perseverative errors correlated with scores Social Media Addiction (20). Finally, a study of inhibitory control mechanisms together with event-related potentials (ERPs) using an SNS Go-No Go task showed no performance differences between problematic and non-problematic users (59). However, there was an indication of larger N1 amplitude following SNS images than control images and a larger N2 amplitude and smaller NoGo-P3 amplitude in excessive users. These findings may suggest inefficient allocation and monitoring of resources and problems inhibitory control mechanisms (59). In summary, there is little evidence that problematic SNS use may lead to impaired flexibility of inhibitory control mechanisms, though there may be ERP evidence for late inhibitory control (unsupported by behavioral data).

### Social needs and “fear of missing out”

It has been suggested that problematic social media use has been associated with FoMO in order to serve and compensate for individuals' social needs (60). FoMO mediated the relationship between the fear of a negative and positive evaluation and social use of the smartphone (60). FoMO and withdrawal ratings were higher among participants with 72 h of restricted access to smartphones compared with those without access to smartphones (61). FOMO also predicted excessive smartphone use by female WhatsApp users (62) and it was associated with the use of smartphones by American undergraduate students for social purposes (63). FoMO mediated the relationship between depression and anxiety and the severity of problematic smartphone use. This finding suggests that individuals experiencing social anxiety who desire social contact are likely to use the smartphone as an avoidance mechanism (63).

A survey of college students has found that greater social activity is a positive predictor of addiction to the social media application Snapchat (64). Among young adults who use Facebook, FoMO, dysfunctional cognitions, and distress predicted problematic SNS use (65). FoMO and rumination mediated the connection between social anxiety and problematic Facebook use (66). FoMO also played an important role in increased sensitivity to stress which is associated with neglect and problematic SNS use, and these in turn were associated with negative emotions (67). Finally, FoMO mediated the association between mental health and symptoms of Internet Use Disorder (IUD) (68).

### Attachment

The evidence on the relationship between attachment and problematic SNS use is scarce. The association between anxious attachment and problematic SNS use was mediated by FoMO and online social support, and online social support negatively mediated the association between avoidant attachment and problematic SNS use (69). A recent review has suggested that problematic SNS use negatively correlated with secure attachment and positively correlated with anxious attachment, but there was no clear association with avoidant attachment. Furthermore, the associations between problematic SNS use and attachment were mediated by individual and interpersonal variables (70).

### Personality

Only few studies that have investigated the relationships between personality and problematic SNS use. Emotional stability, extraversion, and conscientiousness predicted Problematic Facebook use, among adolescents (71). Among the big 5 personality factors, agreeableness, conscientiousness, and self-liking negatively correlated with Instagram addiction (72). Furthermore, self-liking partially mediated the association between Instagram addiction with agreeableness and fully mediated the relationship between Instagram addiction with conscientiousness (72). Conscientiousness, Extraversion, Neuroticism, and Loneliness were predictors of Facebook Addiction. Neuroticism but not extroversion, had a positive correlation with problematic SNS use (73). Furthermore, frequency of status updates mediated the association between each personality trait and problematic SNS use. “Likes” mediated the association between extraversion and problematic SNS use and there was no effect for neuroticism (74). Finally, problematic SNS use correlated with the dark triad traits psychopathy, narcissism and Machiavellianism, and emotion dysregulation (75). Furthermore, emotion regulation played an important role mediating the association between dark triad traits and problematic SNS use (75). Specifically to narcissism, vulnerable narcissists reported a stronger preference for online social interactions and higher overall levels of problematic use of SNSs than grandiose narcissists (76). In summary, problematic SNS use has been associated with FoMO, attachment difficulties and certain personality characteristics like conscientiousness, agreeableness and neuroticism. There is contradictory evidence regarding extraversion and some evidence for an association with narcissism.

### Effects of health including sleep

Few studies have explored the effects of problematic SNS use on sleep quality and duration in adolescents and young adults. A negative correlation was reported between time spent on screen-based devices and sleep quality and quantity (77). Furthermore, higher rates of insomnia, reduced sleep duration, later sleep onset and problems in sleep efficiency and evening screen time were reported among students who also showed similar patterns of sleep problems together with poorer academic achievements, reduced life satisfaction and depression (78). Among young adults in Italy, problematic SNS use was not directly associated with poor sleep quality and it was mediated by depression and stress (79). A majority of Czech adolescents use SNS before bedtime, 20% eat dinner while using SNS, about a third use SNS continuously, SNS use is associated with alcohol use and parental restriction can reduce problematic SNS use (80). Self-reported screen time and symptoms of withdrawal correlated with problematic SNS use, stressing the addictive properties of SNS use among adolescents (81). Finally, problematic SNS use was linked with male and female sexual difficulties (82). In summary, there is evidence that problematic SNS use is associated with reduced sleep quality and quantity, longer sleeping latency and more sleep disturbance and some evidence for sexual problems.

#### COVID-19 effects on problematic SNS use and longitudinal studies

During COVID-19 social distancing in a period when there were hardly any in-person interactions, problematic SNS use correlated positively with frustration over the need to relate to others as well as depressive symptoms and loneliness (83) and negatively with engagement and wellbeing (84). Problematic SNS use was shown as a predictor of emotional distress during covid-19 (85). Several reviews have outlined the problems of social network use during COVID-19. During lockdown prevalence of problematic social media use in young adults was higher compared to non-lockdown periods (86). Although problematic Internet use is associated with health problems in a minority of young people, the COVID-19 pandemic may have enhanced such use and consequently increased health problems (87). Social distancing and lockdowns have increased negative emotions like stress, anxiety, and depression but it had also been associated with positive aspects like enhanced social connections and the use of entertainment. Although most users of Internet technology made an adaptive use of this technology, vulnerable individuals were at risk of developing problematic use of the Internet and require support and guidance (88).

Recently, there is an increasing number of longitudinal studies on problematic social media use, some of them during COVID-19 pandemic with some conflicting results. A reciprocal relationship was reported between the level of problematic SNS use and anxiety over 9 months among Hong Kong and Taiwanese students (89). Increased insomnia and problematic SNS use was reported after 3 month follow-up among Iranian adolescents (90). During three waves of COVID-19 over 6 months in China, problematic social media use was associated with problematic smartphone use, and problematic social media use was associated with an increase in depression and anxiety (91). Although higher levels of problematic smartphone use were not related to greater psychological distress before the COVID-19 outbreak, this prospective relationship became significant during the COVID-19 outbreak in school children in China (92). Chinese schoolchildren spent more time on the smartphone and social media, but not gaming during the school suspension compared to before the outbreak of COVID-19 and those who were highly engaged with Internet-related activities showed an increased level of psychological distress especially during school suspension (93). During COVID-19, problematic use of Internet-related activities in Chinese school children were increased among low and moderate users of the Internet, but it has surprisingly declined among participants with high usage of the Internet (94). A 6-month longitudinal study of Taiwanese students has surprisingly shown that problematic social media use correlated with higher physical activity (95). Finally, a 3-month longitudinal study among Hong Kong students has expectedly shown that social media use negatively correlated with physical activity and sleep quality (96).

### Brain imaging

The go/no-go paradigm was used in 20 Facebook users who responded to Facebook and traffic sign control stimuli in functional MRI (97). There was a positive correlation between Facebook go trials and addiction scores but there was no association between inhibitory mechanisms and addiction scores. These findings have indicated that Facebook addiction shares some neural features with substance and gambling addictions, related to inhibitory-control brain mechanisms (97). Problematic SNS use was associated with reduced gray matter volumes in the amygdala but not in the nucleus accumbens in twenty social network site (SNS) users (98). The authors have argued that these alterations indicate impulsivity thus resemble other addictions. Furthermore, they have found a normal gray matter volume in the Anterior Cingulate Cortex (ACC) which suggests unimpaired inhibition mechanisms (98). Montag (99) have recorded actual Facebook use of 62 participants on their smartphones over the course of 5 weeks and they have reported that higher daily frequency of checking Facebook on the smartphone correlated with smaller gray matter volumes of the nucleus accumbens, suggesting that Facebook use has rewarding effects on the brain. An MRI study of social media users showed a negative correlation between gray matter volume of the insula with problematic SNS use symptoms that was mediated by delay discounting, an indicator of impulsivity (100). Symptom severity of problematic SNS use correlated with attentional impulsivity but not with executive function or inhibitory control of SNS-related cues (101). Finally, a study has investigated social anxiety-related inhibitory control comparing individuals who are addicted to gaming, problematic SNS users, and control participants. They have used a Go/no Go task with emotional words and the Emotional Stroop Task in fMRI (102). IGD participants showed impulsivity, social anxiety and impaired emotional competence, however there were no between group differences in performance of both tasks. During interference of socially anxious words there was decreased middle and superior temporal gyrus activity in gaming addicted participants compared with problematic SNS users (102). In summary, there is evidence that problematic SNS use is associated with impaired inhibitory mechanisms and reduced gray matter in several brain structures such as the amygdala, nucleus accumbens and the Insula. There is some evidence for impulsivity in these users but it is based on correlation and not on group differences.

### Intervention

A short-term abstinence intervention program based on Cognitive Behavior Therapy (CBT), has treated 65 clients with problematic SNS use. They had sessions of over 2 h breaks from social media for 2 weeks, compared with a control group that has used social media as usual. Intervention had a positive effect on emotional wellbeing, behavioral and cognitive function during abstinence and afterwards. These findings suggest that CBT-based short-term abstinence intervention can be useful to improve problematic SNS use (103). There is also preliminary evidence that “Social Media Detoxification” among university students can increase positive mood, reduce anxiety and improve sleep during and immediately after detoxification (104).

## Discussion

Problematic SNS use is associated with potentially harmful behaviors such as loss of control over daily life activities, low self-esteem, anxiety, loneliness and depression. The studies reviewed so far have consistently shown evidence of comorbidity with psychiatric disorders such as depression, anxiety, OCD, stress and ADHD resembling what was reported by Weinstein and colleagues in adolescents and young adults diagnosed with Internet and Gaming Disorder and with excessive smartphone use (105, 106). Other emotional factors included ruminating thoughts, aggression and problems in emotion regulation. This similarity is not surprising, given the strong correlation between measures of Internet addiction and problematic SNS use that was found through these studies. Both conditions are characterized by the loss of cognitive and emotional control, which is associated with impairment in family function and in relationships with friends and low self-esteem. Both conditions also share increased levels of depression, anxiety, and stress and socializing is an important motivation in video game play (14). There are differences between problematic SNS use and Internet addiction. The motivation to use social media can be in order to obtain external social rewards such as “likes,” to make social comparisons and to have connection with a large group of other people rather than enjoyment or subjective wellbeing (54, 55). IGD is closely associated with sensation and novelty seeking (105), impulsivity, enhanced sensitivity to reward and impaired cognitive control (107). There is little evidence for impaired cognitive control and impulsivity in problematic SNS use. Furthermore, there is some evidence that problematic SNS use is characterized by deficits in emotion regulation, for example, reduced striatal activation during self-reflection compared to during ideal reflection while performing on a self-retrieval task in fMRI (108). This is compatible with recent evidence for low self-reflection among problematic SNS users (51).

There is little research on problematic SNS use and personality factors, several studies have found a connection with neuroticism agreeableness and conscientiousness and there are conflicting results regarding the association with extraversion. There is little evidence for impairment in executive cognitive function such as mental flexibility and inhibitory control. There is some ERP evidence of inefficiency in resource allocation and inhibitory control. Due to the lack of difference in behavioral performance this evidence should be interpreted with caution. Also, very few health problems have been reported in problematic SNS use, mainly related to sleep quality and duration. There are few studies on sex differences in problematic SNS use. Turel (109) have shown that the negative association between SNS addiction symptoms and wellbeing is enhanced by neuroticism, and that this enhancement is stronger for women than for men.

There are very few studies investigating whether problematic SNS use meets the main components of “behavioral addiction,” namely; salience, mood modification, tolerance, withdrawal, conflict and relapse (110). Two experiments have used a cue-reactivity paradigm to investigate craving in problematic SNSs use (111). They have found that SNS-related word clues induced craving and excitability in problematic SNS users. Furthermore, craving induced by an image clue was significantly higher than the craving induced by a word clue (111). A recent study has shown that Facebook-related cues elicited larger ERP positivity than other stimuli in Facebook users and that craving correlated with lower later positivity to pleasant and unpleasant cues (112). These findings indicate that Facebook-related cues and craving are given attention priority over other emotions (112). Cue-elicited urges to use SNS correlated with excessive and problematic SNS use (113). Desires and urges to use SNSs (wanting) were dissociated from enjoyment and pleasure (liking) related to SNSs, suggesting that similarly to drug addiction, wanting was more predictive than liking to the intensity and problematic SNS use (113).

There is further evidence for selective attention to SNS cues and deficient decision-making in problematic SNS users. Problematic SNS users who performed on a visual dot probe task, showed attentional bias for SNS-related images, a mechanism that is common to addictive disorders (114). Problematic SNS users also show disadvantageous decision making which was indicated by high self-disclosure posting in SNS sites while neglecting long-term risks (115). There was also evidence that problematic SNS use was associated with impaired decision–making indicated by taking more risk-taking decisions on the Iowa gambling task similarly to individuals with behavior or substance use disorder (116). However, the study reported a negative correlation between Facebook addiction scores and performance on the IGT over the last block of 20 trials but not in earlier blocks of trials. This is weak evidence of impulsivity since it is based on correlation and not on significant group differences in risky decision-making. These studies support the argument that problematic SNS use, similarly to IGD and other behavioral addictions, has the components of craving, selective attention to salient stimuli and impaired decision making. However, they fall short of a full validity of problematic SNS use as a distinct behavioral addiction. Finally, ADHD symptoms have led to increased stress and decreased self-esteem and it has been suggested that together with ADHD, they facilitated cravings to use social network while driving (117).

Few brain imaging studies have investigated the neural correlations of problematic SNS use. There is some evidence for reduced gray matter volume in the amygdala, but not in the ACC and there are conflicting results about gray matter volume in the nucleus accumbens. Several studies have suggested that similar to drug and other behavioral addictions, impulsivity and impaired inhibitory control mechanisms relate to problematic SNS use, but the evidence is mainly based on correlations than actual group differences. There is also little evidence that suggests that brain mechanisms in response to emotional stimuli are different between IGD participants who are addicted to games compared with problematic SNS users. Finally, very few studies on treatment and abstinence, have suggested that CBT based treatment can ameliorate problematic SNS use.

There are several ways of handling the emotional and social problems associated with problematic social media use. First, parents can restrict the use of social media to several hours a day, in particular during bedtime, meals and sports. Especially bedtime is important since social media use has negative effects on sleep quality and quantity. Secondly, alternative activities like indoor and outdoor sport activity and social activity with family should be encouraged. Educational programs at school should be encouraged especially about the negative emotional effects of social media use like loneliness in order to increase awareness to the problem. Educational efforts should be made to deal with the emotional problems associated with excessive social media use. Adolescents should be encouraged not to use social media when they feel “down” or depressed since it can exacerbate their emotional state and also to avoid social comparisons since social medial does not often reflect real life and how to deal with FoMO that is known to exacerbate loneliness and negative emotions.

Finally, problematic social networking use may be associated with self-stigma. Recent studies assessing the problematic social networking use and self-stigma among people with mental illness have shown an association between problematic social networking use and self-stigma. For example, people with substance use disorders were found that their problematic use may lead to their self-stigma (118, 119). It is useful to investigate further this important association.

## Limitations

One of the major limitations in studies of problematic SNS use, similar to Internet and Gaming Disorder and excessive smartphone use is that they are mainly cross-sectional studies without baseline measures and they rely on relationship between structural and functional changes in the brain and subjective measures. These relationships do not provide any proof that problematic SNS use affects the development of the adolescent or adult brain. Factors that mediate such associations tend to be educational, cognitive, emotional, and social in nature. Methodological considerations also include age (e.g., use by adolescents and students), and lack of comparison with substance use disorder. Finally, very few studies have considered sex differences in cognitive and brain function in problematic SNS users.

## Summary

Easy access to the smartphone enables users to connect to social media and social networks. Unfortunately, in some users this can lead to excessive use and may have negative effects on mental health, especially social anxiety and depression. Problematic SNS use affects sleep quality and quantity as well as altered emotional communication patterns, and FOMO. These characteristics should send an alarm signal to clinicians and educators to investigate problematic SNS use particularly in children and adolescents. Gaps in our knowledge- more research is necessary on the cognitive and brain changes associated with problematic SNS use, on personality and sex-differences and treatment. Finally, problematic SNS use correlates strongly with Internet addiction; hence, the similarity in cognitive, emotional, and social consequences. The evidence so far does not support the inclusion of problematic SNS use as a clinical diagnosis as a behavioral addiction but rather as a type of Internet and Gaming Disorder.

## Author contributions

The author confirms being the sole contributor of this work and has approved it for publication.
